# *Streptococcus pneumoniae* favors tolerance via metabolic adaptation over resistance to circumvent fluoroquinolones

**DOI:** 10.1128/mbio.02828-23

**Published:** 2024-01-09

**Authors:** Tina H. Dao, Haley Echlin, Abigail McKnight, Enolia S. Marr, Julia Junker, Qidong Jia, Randall Hayden, Tim van Opijnen, Ralph R. Isberg, Vaughn S. Cooper, Jason W. Rosch

**Affiliations:** 1 Department of Infectious Diseases, St. Jude Children’s Research Hospital, Memphis, Tennessee, USA; 2 Nationales Referenzzentrum für Streptokokken Abteilung Medizinische Mikrobiologie, Universitätsklinikum RWTH Aachen, Aachen, Germany; 3 Department of Pathology, St. Jude Children’s Research Hospital, Memphis, Tennessee, USA; 4 Broad Institute of MIT and Harvard, Cambridge, Massachusetts, USA; 5 Deptartment of Molecular Biology and Microbiology, Tufts University School of Medicine, Boston, Massachusetts, USA; 6 Center for Evolutionary Biology and Medicine, University of Pittsburgh, Pittsburgh, Pennsylvania, USA; McMaster University, Hamilton, Ontario, Canada; Northeastern University, Boston, Massachusetts, USA

**Keywords:** *Streptococcus pneumoniae*, antibiotic resistance, virulence

## Abstract

*Streptococcus pneumoniae* is a major human pathogen of global health concern and the rapid emergence of antibiotic resistance poses a serious public health problem worldwide. Fluoroquinolone resistance in *S. pneumoniae* is an intriguing case because the prevalence of fluoroquinolone resistance does not correlate with increasing usage and has remained rare. Our data indicate that deleterious fitness costs in the mammalian host constrain the emergence of fluoroquinolone resistance both by *de novo* mutation and recombination. *S. pneumoniae* was able to circumvent such deleterious fitness costs via the development of antibiotic tolerance through metabolic adaptation that reduced the production of reactive oxygen species, resulting in a fitness benefit during infection of mice treated with fluoroquinolones. These data suggest that the emergence of fluoroquinolone resistance is tightly constrained in *S. pneumoniae* by fitness tradeoffs and that mutational pathways involving metabolic networks to enable tolerance phenotypes are an important contributor to the evasion of antibiotic-mediated killing.

**IMPORTANCE:**

The increasing prevalence of antibiotic resistant bacteria is a major global health concern. While many species have the potential to develop antibiotic resistance, understanding the barriers to resistance emergence in the clinic remains poorly understood. A prime example of this is fluroquinolone resistance in *Streptococcus pneumoniae*, whereby, despite continued utilization, resistance to this class of antibiotic remains rare. In this study, we found that the predominant pathways for developing resistance to this antibiotic class severely compromised the infectious capacity of the pneumococcus, providing a key impediment for the emergence of resistance. Using *in vivo* models of experimental evolution, we found that *S. pneumoniae* responds to repeated fluoroquinolone exposure by modulating key metabolic pathways involved in the generation of redox molecules, which leads to antibiotic treatment failure in the absence of appreciable shifts in resistance levels. These data underscore the complex pathways available to pathogens to evade antibiotic mediating killing via antibiotic tolerance.

## INTRODUCTION

*Streptococcus pneumoniae* is a major cause of morbidity and mortality particularly in young children and elderly populations (1). Annually, about half a million deaths among children younger than the age of 5, the immunocompromised, and the elderly result from pneumococcal disease worldwide (2). There has been a worldwide increase in the number of cases of *S. pneumoniae* resistant to multiple classes of antibiotics, including folate inhibitors (trimethoprim and sulfamethoxazole), cell-wall synthesis inhibitors (penicillin), and protein synthesis inhibitors (azithromycin, doxycycline, tetracycline, and minocycline) (3). Unlike macrolide and beta-lactam resistance, there have been paradoxical observations in the case of fluoroquinolone resistance. Globally, fluoroquinolones are one of the most frequently prescribed antibiotics, however only limited cases of highly fluoroquinolone-resistant pneumococcal isolates have been reported (Table S1) (4–6). Unlike the relatively widespread macrolide and to a lesser extent beta-lactam resistance, fluoroquinolone resistance in *S. pneumoniae* remains infrequent with only 1–2% of pneumococcal isolates in the United States being resistant (4, 7). This low prevalence is also observed for another bacterial respiratory pathogen, *Haemophilus influenzae,* but contrasts with high prevalence observed in *Escherichia coli* (8–10). These data suggest that the genetic pathways to resistance may be relatively inaccessible or there may be fitness costs imposed by acquiring fluoroquinolone resistance in *S. pneumoniae* and these pathways may be species or niche dependent.

Fluoroquinolones (such as ciprofloxacin, levofloxacin, and moxifloxacin) inhibit topoisomerase IV (ParC and ParE) and gyrase A (GyrA), which are responsible for unwinding supercoiled DNA, thereby halting DNA replication and resulting in cell death (11). Mutations acquired via recombination with resistant strains or by *de novo* mutation in either of these two genes can confer high-level resistance to fluoroquinolones in *S. pneumoniae* (12). The propensity of the pneumococcus for genetic exchange permits antibiotic resistance determinants to rapidly spread amongst strains which, in theory, would allow for widespread dissemination of fluoroquinolone resistance. Despite this capacity for rapid spread of resistance, pneumococcal strains harboring mutations conferring fluoroquinolone resistance remain relatively rare.

Often overshadowed by the prominent phenomenon of antibiotic resistance, tolerance is becoming increasingly recognized as being important mechanisms underlying antibiotic treatment failure and recalcitrant infections (13). Tolerance refers to the ability of the bacteria to withstand the lethal actions of bactericidal antibiotics with markedly reduced bactericidal kinetics following antibiotic exposure without significant increases in the minimum inhibitory concentration (MIC) (14). While antibiotic resistance is readily detected by traditional clinical MIC assays and thus has been extensively studied and characterized, tolerance is often overlooked and undetected. Although antibiotic kill kinetics (or time-kill curves) have been conducted to detect antibiotic tolerance, such assays are laborious to undertake as part of routine diagnostics (15). Despite these obstacles, antibiotic tolerance has been extensively reported in both Gram-negative and Gram-positive bacteria and serves as a potential avenue for the subsequent development of high-level resistance (16, 17). Tolerance pathways include stress response, metabolic regulation, transcriptional regulation, efflux/influx regulation, and genes involved in metabolism, transport, and regulation of gene expression (18–20). This underscores the multiple available mechanisms by which pathogens can become tolerant and evade antibiotic killing in the absence of resistance.

Antibiotic treatment failure, whereby initial antibiotic therapy fails to effectively clear the infection, can result in prolonged and recalcitrant infections and poorer clinical outcomes (21–25). For bacterial nosocomial pneumonias, initial antibiotic treatment failure has been reported as high as >70% of patients and, even when resistant organisms are not present, the mortality rate for hospitalized patients can be greater than 50%. Although not detected by traditional testing, these bacteria recalcitrant to antibiotic killing are increasingly recognized as important contributors to antibiotic treatment failure (26–29). A key factor in whether antibiotic tolerance and resistance emerge and spread within a population is the fitness costs of these mutations, such as decreased replication speed (30, 31). While such mutations enable the bacteria to survive under antibiotic pressure, they often render the target enzymes suboptimal in the absence of antibiotics and the bacteria can be effectively outcompeted following cessation of antibiotic pressure. It is strains that obtain mutations that engender the capacity to survive antibiotic exposure without conferring corresponding fitness tradeoffs that represent the greatest concern for the spread of antibiotic resistance resulting in treatment failure.

In our current study, we sought to gain insight into the constraints of fluoroquinolone resistance and the means to circumvent these constraints in *S. pneumoniae*. We found that fluoroquinolone resistance mutations in either of the genes encoding the canonical topoisomerases, *gyrA* or *parC*, imparted a high fitness cost during infection, with only double mutants retaining the capacity to cause invasive disease. Moreover, experimentally evolved strains subjected to repeated fluoroquinolone treatment failed to acquire these mutations and evolve resistance, but rather favored the emergence of tolerance that contributed to antibiotic treatment failure. Genetic analysis of the evolved, tolerant isolates implicated loss-of-function mutations that abrogated hydrogen peroxide production as a potential underlying mechanism. This was confirmed with targeted deletions and via chemical inhibitors, which demonstrated that reduction of oxygen radical production conferred tolerance to fluoroquinolones both *in vitro* and *in vivo*. This phenotype was linked to reduced DNA damage during antibiotic treatment, suggesting that the reduction of oxidative stress is a critical mediator of the bactericidal activity of fluoroquinolones. These data indicate that *S. pneumoniae* can evade antibiotic-mediated killing via inactivation of specific metabolic networks to enable tolerance phenotypes instead of the development of resistance mutations which come at a high fitness cost.

## RESULTS

### Fluoroquinolone resistance development during experimental evolution

**Fig 1:**
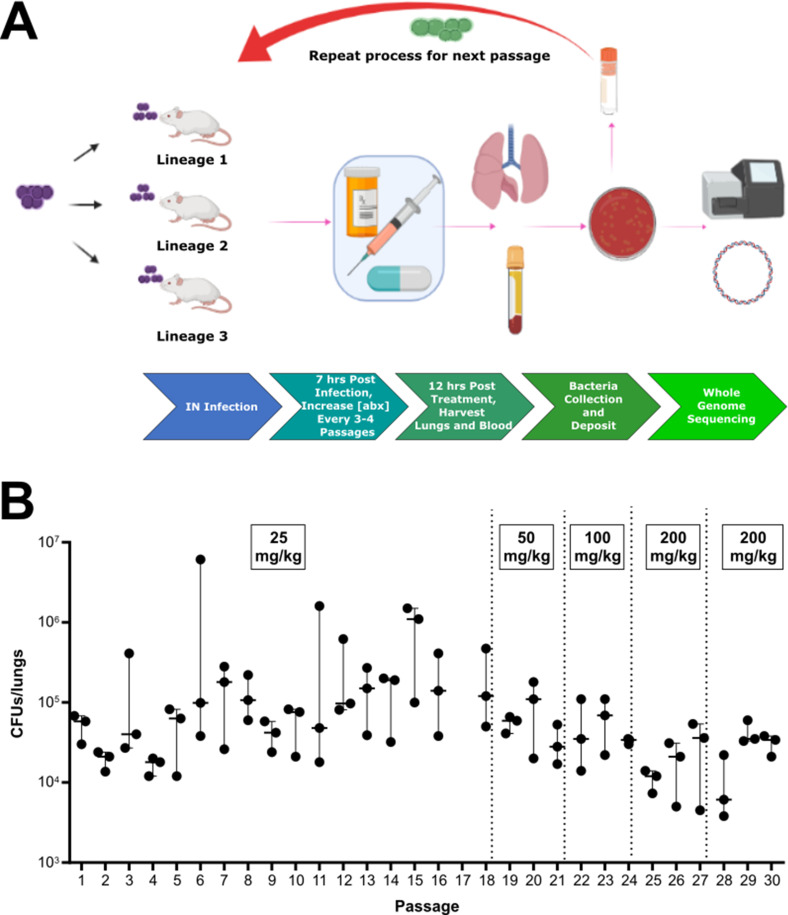
Experimental evolution of *S. pneumoniae* in response to levofloxacin during infection. Schematic diagram of *in vivo* passaging workflow. Three mice, representing three lineages, were intranasally infected with TIGR4 and, after 6 h of infection, mice were treated with levofloxacin. Twelve hours after levofloxacin treatment, bacteria were collected from lungs of infected mice and recovered on media plates following overnight incubation. Frozen aliquots from each passage served as the inoculum to infect the subsequent group of mice for each lineage. Created with BioRender.com (**A**). After each passage under levofloxacin pressure, bacterial burden in the lungs was enumerated. Dosage of levofloxacin ranged from 25 to 200 mg/kg as indicated. Each data point represents an individual mouse and bars represent median with range (**B**). Significance was observed comparing CFUs over all passages via Krukal-wallis one-way ANOVA (*P* = 0.0079) but no significance was observed comparing each passage to passage 1 via Mann-Whitney using Prism 6.

Although the prevalence of fluoroquinolone utilization remains high, pneumococcal resistance remains relatively low compared to other antibiotics (Table S1). We first attempted to determine whether clinical observations for the emergence of resistance could be recapitulated in a murine model of infection and to determine what mutations could arise that would permit resistance. To search for mutations associated with fluoroquinolone resistance operative during infection, we utilized a murine model of *in vivo* experimental evolution (Fig. 1A). The wild-type TIGR4 underwent 30 passages in three independent lineages in BALB/c mice under increasing levofloxacin antibiotic dosage (Fig. 1B). Whole-genome sequencing analysis of each experimentally evolved population collected from the final passage indicated the absence of on-target mutations in either *gyrA* or *parC*. In concordance with this observation, minimal shifts in MIC were observed, where the levofloxacin MIC of TIGR4 is 1 µg/mL (Tables S23; Fig. S1). These results indicated that the development of resistance remains absent despite repeated antibiotic exposure, similar to clinical observations. This result contrasted with *in vitro* evolved populations, whereby shifts in resistance were achieved, with relatively minor shifts in response to levofloxacin (Fig. S2A) and larger shifts above the MIC observed with ciprofloxacin (Fig. S2B).

### Acquisition of fluoroquinolone resistance by recombination

**Fig 2:**
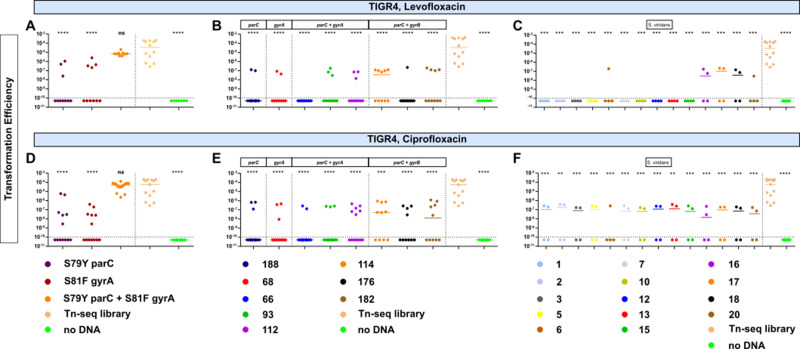
Transformation efficiency for fluoroquinolone resistance determinants in TIGR4. Transformation efficiency of fluoroquinolone resistance determinants in TIGR4 (serotype 4). Resistant colonies were recovered on plates supplemented with either 2 µg/mL levofloxacin (**A–C**) or 4 µg/mL ciprofloxacin (**D–F**). DNA used for transformation included either PCR fragments encoding the respective mutations (**A, D**), genomic DNA of *S. pneumoniae* clinical isolates harboring the respective mutations (**B, E**), or genomic DNA of *S. viridans* clinical isolates harboring fluoroquinolone resistance (**C, F**). Transformation efficiency was calculated as the ratio of the number of transformants (CFUs/mL) selected on either levofloxacin or ciprofloxacin to the number of total bacteria (CFUs/mL). For all transformations, a Tn-seq library served as a positive control for competence and transformation efficiency with each donor DNA was compared to that of the TN-seq library via Mann-Whitney using Prism 6. **P < 0.01, ***P < 0.001, and ****P < 0.0001. Each data point represents an individual biological replicate and bars represent median. Dashed line represents limit of detection.

As antibiotic resistance in the naturally competent *S. pneumoniae* is oftentimes acquired via recombination with other strains or species (32), we next sought to ascertain the relative recombination frequency of fluoroquinolone resistance. Transformation efficiency of the wild-type TIGR4 with PCR fragments encoding point mutations in either *gyrA* (S81F) or *parC* (S79Y), was measured following selection on either levofloxacin (Fig. 2A) or ciprofloxacin (Fig. 2D). These mutations were selected as they have been shown to confer high-level resistance to fluoroquinolones (33–37). TIGR4 transformation with either single point mutation occurred at a much lower frequency compared to an irreverent antibiotic resistance mechanism (Tn-seq library containing spectinomycin resistance) which consistently transformed at an order of magnitude greater efficiency. Negative controls lacking DNA were assayed in parallel to account for spontaneous resistance mutations (Fig. 2A and D). To test whether this low transformation efficiency was strain or serotype-specific, we transformed additional *S. pneumoniae* strains including D39 serotype 2 (Fig. S3A and D) and CDC001 serotype 9V (Fig. S3G and J) and observed similar transformation patterns, indicating recombination of fluoroquinolone resistance was relatively inefficient and not due to a reduced competent state.

Compensatory mutations have been shown to ameliorate fitness costs of other mutations, allowing both to fix in the population (38, 39). As such, we tested if fluoroquinolone resistance occurred more readily via transformation with DNA from strains that harbored both on-target and compensatory mutations (Table S2). The transformation efficiency of TIGR4, D39, and CDC001 using gDNA from fluoroquinolone-resistant strains remained extremely low, though using gDNA containing mutations in *parC* and *gyrA* occurred more readily (Fig. 2B and E; Fig. S3B, E, H, and K). We also explored another major genetic reservoir of antibiotic resistance determinants for *S. pneumoniae,* the viridans group streptococci (40). Transformation with genomic DNA of fluoroquinolone-resistant viridans group streptococci (Table S4) still rarely produced resistant strains. The TIGR4 strain had a relatively high efficiency of 10^−7^ when transformed with isolates 16, 17, and 18, which encoded double point mutations in *parC* and *gyrA*, under levofloxacin (Fig. 2C) or when transformed with all isolates under ciprofloxacin (Fig. 2F). No transformants were observed in the D39 and CDC001 strains (Fig. S3C, F, I, and L). The lack of observable transformants may be due to strain-specific genetic or capsular effects on recombination frequency (41, 42). These results further corroborate barriers for the acquisition of fluoroquinolone resistance determinants via recombination in *S. pneumoniae*.

### Fitness consequence of fluoroquinolone resistance mutations

**Fig 3:**
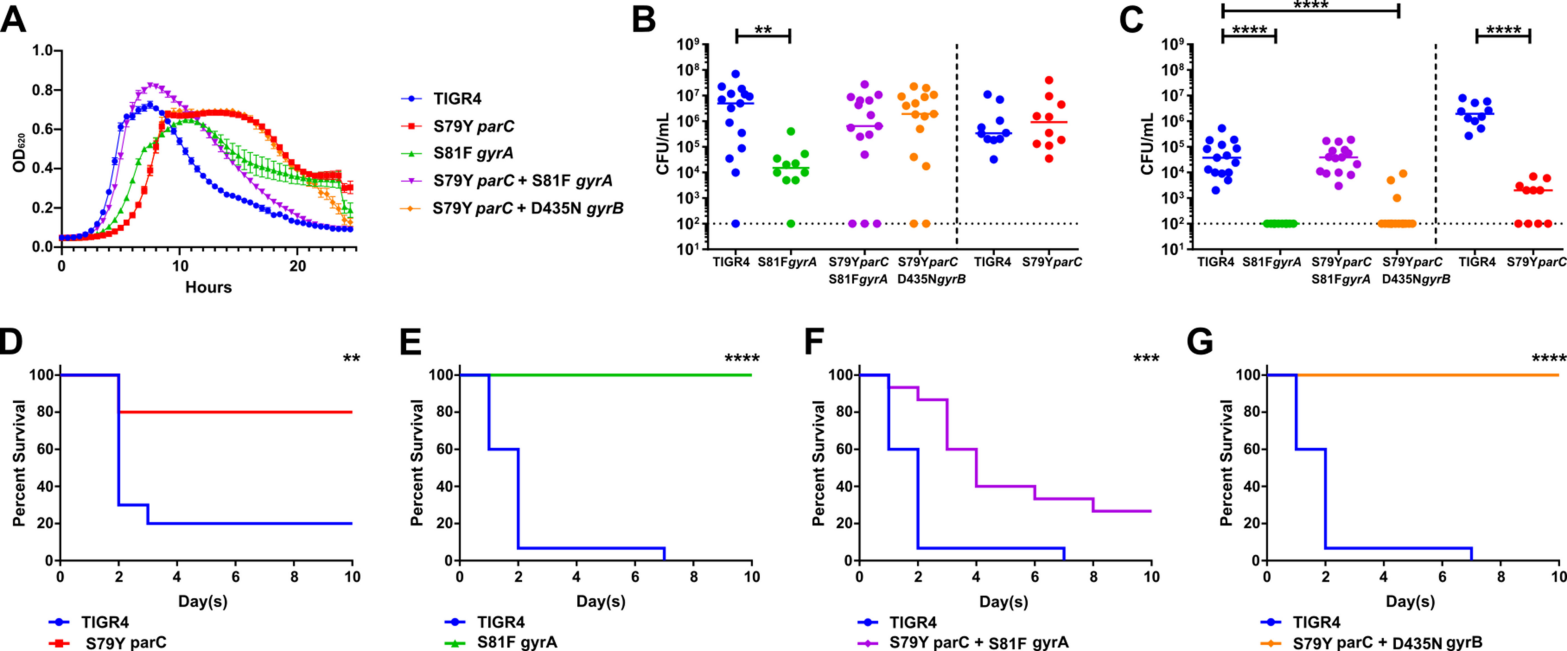
Murine *in vivo* fitness of individual fluoroquinolone resistance mutations during colonization and invasive disease. The respective mutants conferring fluoroquinolone resistance were initially assayed for growth *in vitro* in semi-chemically defined media (**A**). Mice (*N* = 10–15) were infected intranasally with the TIGR4 wild-type or the isogenic fluoroquinolone mutants. Nasal lavage at 24 h (**B**) post-challenge was used to ascertain relative bacterial colonization burden. Invasive potential was assayed via blood titers at 24 h (**C**) post-challenge. Infected mice were followed for 10 days for relative survival between the wild-type and respective point mutants (**D–G**). For panel **A**, data represent nine biological replicates with the mean and SEM plotted. Growth curves of mutants were compared to that of TIGR4 via two-way ANOVA, repeated measures; all comparisons were significant. ****P < 0.0001. For panels **B–G**, each data point represents an individual mouse, bars represent median, and dashed line represents limit of detection. The bacterial burden data were compared to that of the wild-type strain via Mann-Whitney and survival data were analyzed with Mantel-Cox log-rank tests in Prism 6. *P < 0.05, **P < 0.01, and ****P < 0.0001.

We hypothesized that fluoroquinolone resistance was not developed during *in vivo* evolution due to fitness defects of the mutations in *gyrA/parC* conferring resistance. To test this hypothesis, we generated a panel of single- and double-point mutants including S79Y *parC*, S81F *gyrA*, S79Y *parC*/*gyrA*, and S79Y *parC*/D435N *gyrB*. These mutations have been shown previously to confer high-level resistance to fluoroquinolones in clinical isolates (43–46). The growth kinetics of the mutants indicated that S79Y *parC* and S81F *gyrA* demonstrated a slight but marked delay in growth compared to the wild-type TIGR4 (Fig. 3A), while demonstrating shifts in MIC (Tables S2 and S3; Fig. S4). Interestingly, the double mutant S79Y *parC*/S81F *gyrA* had similar growth rate to that of the wild type, while the double mutant S79Y *parC*/D435N *gyrB* had similar growth pattern to that of the S79Y *parC* mutant (Fig. 3A).

We next determined the *in vivo* fitness of the single and double mutants by determining their virulence in a murine model of intranasal infection. Compared to the wild-type TIGR4, the single mutants in either *gyrA* or *parC* resulted in severe fitness defects *in vivo* (Fig. 3B–G). Unlike the single mutant in *gyrA*, the single mutant in *parC* was able to colonize the nasopharynx as effectively as the wild type (Fig. 3B). Although there was a log decrease in bacterial burden compared to the wild type, the double point mutant S79Y *parC*/S81F *gyrA* still colonized the nasopharynx (Fig. 3B). Neither of the single mutants in *gyrA* or *parC* was detected at high levels in the bloodstream, indicating that they were not able to disseminate and/or survive in the bloodstream to cause invasive disease (Fig. 3C). When both the *gyrA* and *parC* mutations were present, fitness was restored to the equivalent to that observed in the wild type in the blood, whereas the *parC/gyrB* double mutant was attenuated similar to the *parC* single mutant (Fig. 3C).

In addition to measuring bacterial burden in the nasopharynx and blood, we monitored infected mice for survival for 10 days (Fig. 3D through G). Mice infected with the single mutants in *gyrA* or *parC* and the double mutant S79Y *parC*/D435N *gyrB* showed a significant increase in survival compared to those infected with TIGR4 (Fig. 3D, E and G). Mice infected with the double mutant S79Y *parC*/S81F *gyrA* displayed significantly delayed mortality, but overall survival was similar to the wild type (Fig. 3F). Of note, two variants of the S81F *gyrA* were observed, including colonies that produced reduced levels of capsule and those that produced wild-type (normal) levels of capsule. However, the presence of capsule did not dramatically impact the overall reduction in the fitness of the *gyrA* mutation as both variants had reduced growth rate and survival in the murine model (Fig. S5). These data suggest that the individual mutations associated with fluoroquinolone resistance in either *gyrA* or *parC* impart significant fitness tradeoffs during infection that restrict their emergence *in vivo*, demonstrating an important evolutionary barrier to the acquisition of fluoroquinolone resistance in *S. pneumoniae*.

### Levofloxacin and host pressure favor development of tolerance over resistance

**Fig 4:**
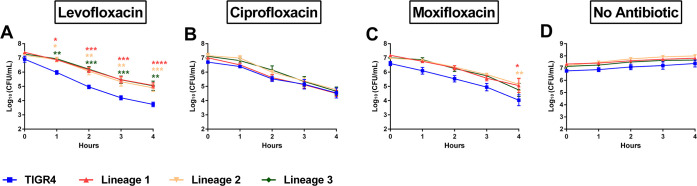
Experimentally evolved isolates of *S. pneumoniae* exhibited tolerant phenotype to levofloxacin. The TIGR4 wild type and the final populations from the three independent lineages evolved in mice were assayed for time-kill kinetics in response to 4×MIC of different fluoroquinolone antibiotics—4 µg/mL levofloxacin (**A**), 8 µg/mL ciprofloxacin (**B**), 0.48 µg/mL moxifloxacin (**C**), and no antibiotic (**D**). Cell numbers were calculated and plotted as log_10_ (CFU/mL). Data represent at least four independent biological replicates with the mean and SEM plotted. For each antibiotic concentration, log_10_ (CFU/mL) of TIGR4 was compared to that of each lineage using two-way ANOVA multiple comparisons, comparing the mean of each time point in Prism 6. *P < 0.05, **P < 0.01, ***P < 0.001, and ****P < 0.0001. No significant difference was observed for any time point without a designated *P* value.

Given that increasingly higher dosages of levofloxacin failed to clear the infections in the absence of MIC shifts, we next sought to determine the tolerance phenotypes of the evolved isolates. Tolerant strains, by definition, can survive transient exposure to antibiotics by having slower kill kinetics than susceptible strains (14). We assayed the antibiotic kill kinetics of three experimentally evolved lineages and observed reduced antibiotic-mediated killing following addition of levofloxacin (Fig. 4A), suggesting that tolerance to levofloxacin emerged in all three independent lineages. We next tested whether the experimentally evolved isolates conferred cross-tolerance to other clinically relevant fluoroquinolones, such as ciprofloxacin (MIC of 2 µg/mL) and moxifloxacin (MIC of 0.12 µg/mL). The isolates exhibited kill kinetics in response to ciprofloxacin and moxifloxacin similar to the wild-type TIGR4 (Fig. 4B and C). When subjected to moxifloxacin, the three evolved lineages demonstrated slight reduced killing at the later time point (Fig. 4C). These data indicate that the evolved tolerance phenotype was specific for levofloxacin and not to other fluoroquinolones. No differences in MIC to levofloxacin, ciprofloxacin, or moxifloxacin were observed in these evolved lineages compared to the wild type via µbroth MIC testing (Fig. S1). These data indicate that levofloxacin tolerance emerged following prolonged *in vivo* passaging under levofloxacin pressure.

**Fig 5:**
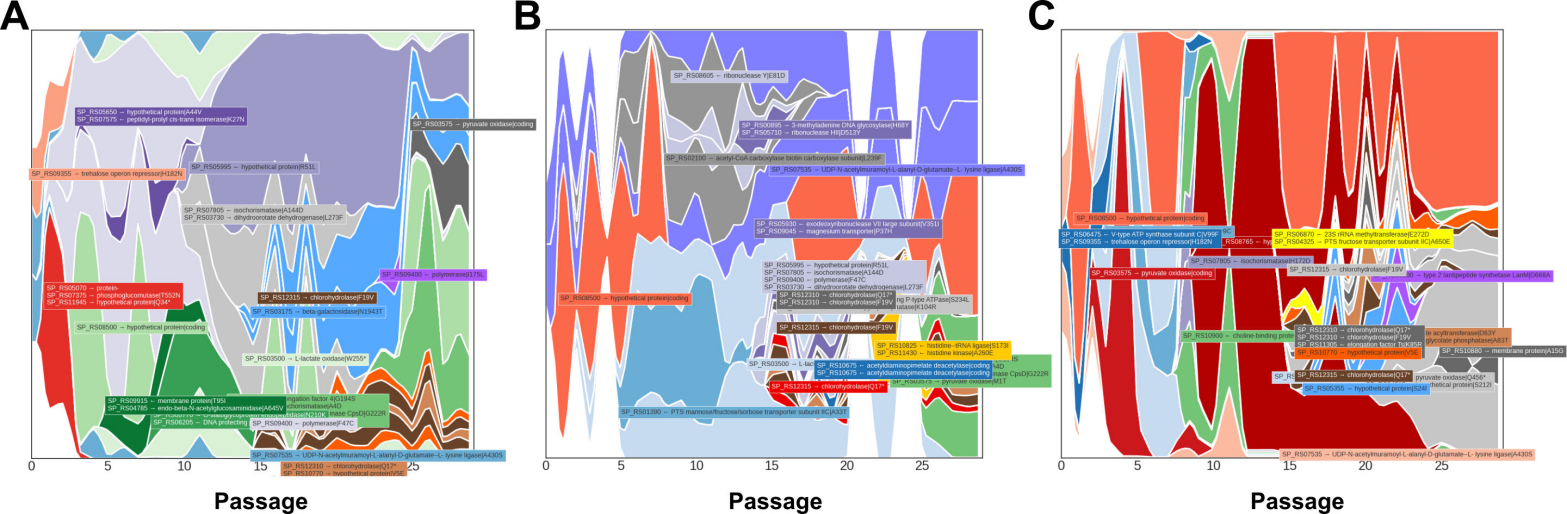
Population analysis of mutations arising during *in vivo* experimental evolution. Muller plots representing relative abundance of selected mutations that arose in multiple lineages during *in vivo* passaging with levofloxacin: Lineage 1 (**A**), Lineage 2 (**B**), and Lineage 3 (**C**). Left panels indicate frequency of mutations of interest for production of hydrogen peroxide, whereas right panels are the entire population dynamics. Red asterisks indicate genotypes highlighted in the adjacent panels to the left.

**Fig 6:**
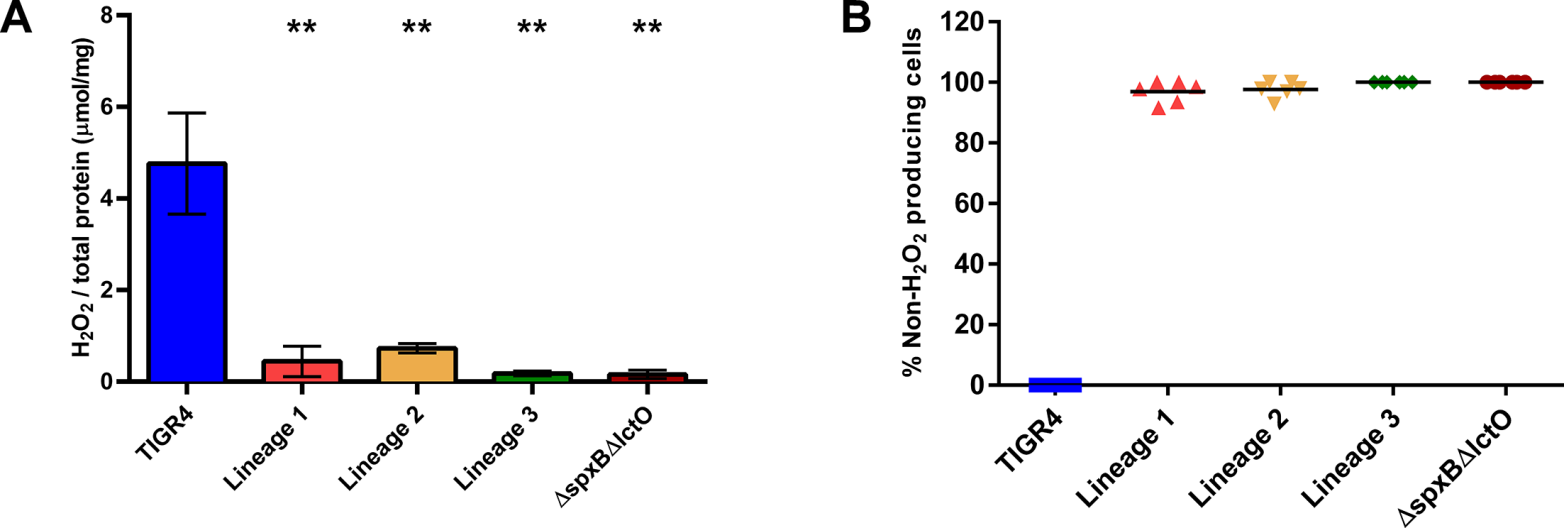
The evolved isolates produced minimal H_2_O_2_. H_2_O_2_ production was measured using two methods: the Amplex Red kit (**A**) and colorimetric agar plates containing horseradish peroxidase and 2,2′-azinobis(3-ethylbenzothiazoline-6-sulphonic acid) (**B**). Strains included the wild-type TIGR4, the final passage of three independent lineages generated through *in vivo* passaging under levofloxacin treatment, and the Δ*spxB*Δ*lctO* double knockout mutant. The ∆*spxB*∆*lctO* double knockout mutant served as the negative control. For the Amplex Red kit, levels were reported as µmoles of hydrogen peroxide per mg of cellular protein, determined via BCA assay. Hydrogen peroxide production of the evolved isolates was compared to that of wild-type TIGR4 via unpaired parametric *t* test in Prism 6. **P value < 0.01. Data represent three biological replicates plotted as mean and SD. For the colorimetric agar plates, the percent of non-hydrogen-producing cells was calculated as the CFU/mL of white colonies divided by the total number of colonies. Each data point represents an individual biological replicate (*N* = 6) and bars represent median.

We next investigated the genetic landscape of the populations that arose during passaging in the murine model (Fig. 1) to identify a genetic basis for the observed tolerance phenotypes, focusing on those mutations that occurred in multiple lineages and in high frequencies in the population. Mutations in isochorismatase (A44D, A4D, and H172D) arose in three independent lineages during *in vivo* evolution with levofloxacin, starting in passages 8–10 and being sustained in the population (Fig. 5). In all lineages, loss-of-function mutations in *lctO* were identified; this gene encodes a lactate oxidase responsible for the conversion of pyruvate to lactate, generating hydrogen peroxide (H_2_O_2_). Later passages revealed that mutations in *spxB* (M1T, Q456*) and chlorohydrolase (Q17*, F19V) emerged and were sustained in the population until the final passage in two lineages (Fig. 5). The missense mutation in the start codon M1T of *spxB* results in a non-functional pyruvate oxidase, which, like lactate oxidase, is involved in pyruvate metabolism and H_2_O_2_ production. These data suggest that alterations in metabolic genes associated with pyruvate metabolism and generation of H_2_O_2_ may be one avenue for evolving tolerance to levofloxacin. Abrogation of H_2_O_2_ production in the evolved populations was subsequently confirmed (Fig. 6), with the wild-type TIGR4 and the ∆*spxB*∆*lctO* double knockout, serving as positive and negative controls, respectively (47).

### Avoidance of redox stress plays a critical role in tolerance to fluoroquinolones

**Fig 7:**
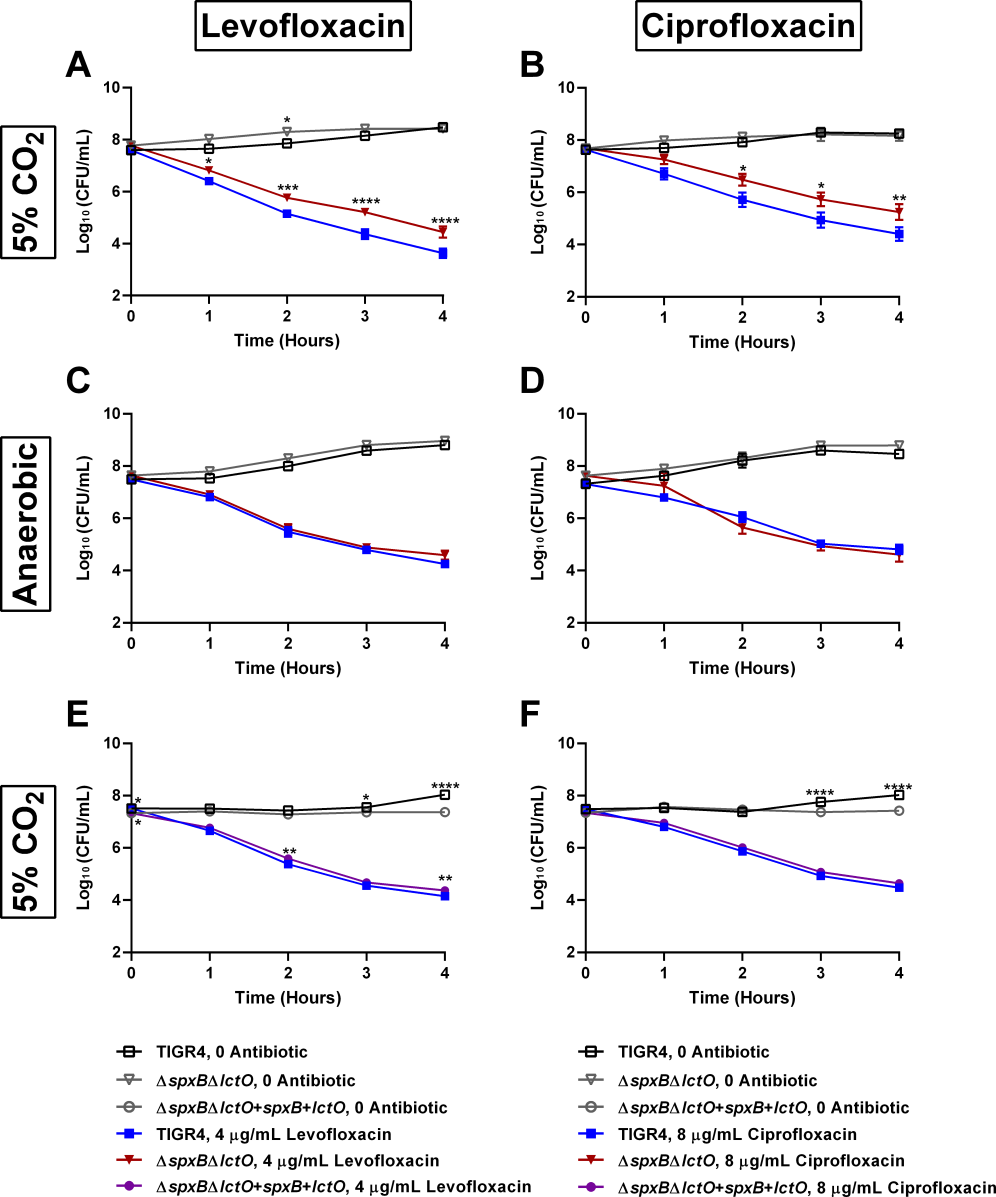
Disruption of endogenous hydrogen peroxide production confers tolerance to fluoroquinolones. The TIGR4 wild-type and the ∆*spxB*∆*lctO* double mutant were assayed for time-kill kinetics in response to different fluoroquinolone antibiotics and levels of oxygen: 4 µg/mL levofloxacin (**A**), 8 µg/mL ciprofloxacin (**B**), 4 µg/mL levofloxacin under anaerobic conditions (**C**), and 8 µg/mL ciprofloxacin under anaerobic conditions (**D**). The complemented ∆*spxB*∆*lctO* double mutant, along with the TIGR4 wild type, was assayed for time-kill kinetics in response to different fluoroquinolone antibiotics: 4 µg/mL levofloxacin (**E**) and 8 µg/mL ciprofloxacin (**F**). Cell numbers were calculated and plotted as log_10_ (CFU/mL). Data represent three independent biological replicates with the mean and SEM plotted. Log_10_ (CFU/mL) of TIGR4, 0 antibiotic was compared to that of ∆*spxB*∆*lctO*, 0 antibiotic using two-way ANOVA multiple comparisons, comparing the mean of each time point in Prism 6. Log_10_ (CFU/mL) of TIGR4, with antibiotic was compared to that of ∆*spxB*∆*lctO*, with antibiotic similarly (**A–D**). Log_10_ (CFU/mL) of TIGR4, 0 antibiotic was compared to that of the complemented ∆*spxB*∆*lctO*, 0 antibiotic and log_10_ (CFU/mL) of TIGR4, with antibiotic was compared to that of the complemented ∆*spxB*∆*lctO*, with antibiotic similarly (**E and F**). *P < 0.05, **P < 0.01, ***P < 0.001, and ****P < 0.0001. No significant difference was observed for any time point without a designated *P* value.

**Fig 8:**
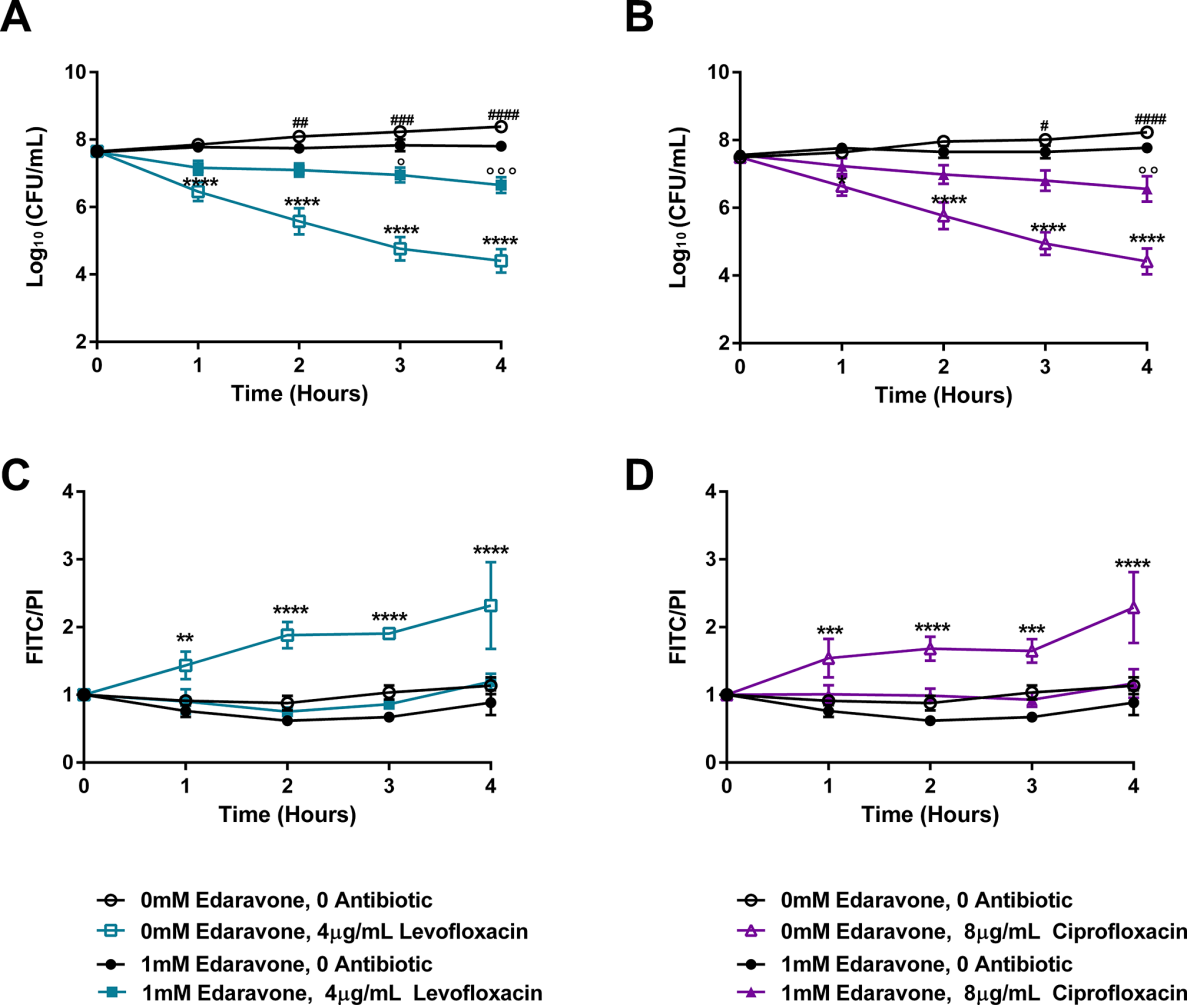
The *S. pneumoniae* treated with free-radical scavenger edaravone demonstrates tolerance to fluoroquinolones and decreased DNA fragmentation. The TIGR4 wild type was assayed for time-kill kinetics upon edaravone supplementation in the presence of 4 µg/mL levofloxacin (**A**) or 8 µg/mL ciprofloxacin (**B**). DNA fragmentation in response to either 4 µg/mL levofloxacin (**C**) or 8 µg/mL ciprofloxacin (**D**) was measured concurrently with the time-kill kinetic experiment. Cell numbers were calculated and plotted as log_10_ (CFU/mL) (**A, B**). Data represent three biological replicates with the mean and SEM (**A, D**) or SD (**C, D**) plotted. Zero millimolar edaravone, 0 antibiotic was compared to that of 1 mM edaravone, 0 antibiotic using two-way ANOVA multiple comparisons, comparing the mean of each time point in Prism 6 (#). Zero millimolar edaravone, 0 antibiotic was compared to that of 0 mM edaravone, with antibiotic (*) and 1 mM edaravone, 0 antibiotic was compared to that of 1 mM edaravone, with antibiotic similarly (°). #,° *P* < 0.05; **, ##, °°*P* < 0.01; ***, ###, °°° *P* < 0.001; ****, #### *P* < 0.0001. No significant difference was observed for any time point without a designated *P* value.

To determine whether metabolic pathways responsible for endogenously produced H_2_O_2_ contribute to the observed tolerance phenotype, we measured kill kinetics of strains or conditions that reduced H_2_O_2_ production. The ∆*spxB*∆*lctO* double mutant, which does not produce H_2_O_2_ (Fig. 6) and has a similar MIC to TIGR4 (Table S3; Fig. S4), demonstrated significantly reduced kill kinetics to both levofloxacin and ciprofloxacin (Fig. 7A and B). As the production of H_2_O_2_ requires the presence of oxygen, we additionally tested the effects of levofloxacin and ciprofloxacin under anaerobic conditions and observed no differences in kill kinetics between the wild-type and the ∆*spxB*∆*lctO* double mutant (Fig. 7C and D). A genetically complemented ∆*spxB*∆*lctO* double mutant, which produced similar levels of H_2_O_2_ as TIGR4 (Fig. S6), demonstrated similar kill kinetics as TIGR4 for both levofloxacin and ciprofloxacin (Fig. 7E and F). Besides the H_2_O_2_ endogenously produced by the pneumococcus, the bactericidal activities of fluoroquinolones may be impacted by free radicals induced by the antibiotic. To test this, we utilized a clinically approved drug (edaravone) that scavenges free radicals in kill kinetic experiments (48, 49). Supplementation of the media with edaravone dramatically reduced the bactericidal activities of both levofloxacin and ciprofloxacin, resulting in over two orders of magnitude reduction in killing following four hours of antibiotic exposure (Fig. 8A and B). As both redox stress and fluoroquinolones can result in DNA damage and fragmentation that contributes to bactericidal activity (50, 51), we next sought to determine whether the protection engendered by edaravone correlated with a corresponding decrease in DNA fragmentation during fluoroquinolone exposure. As expected, treatment with either levofloxacin or ciprofloxacin resulted in elevated DNA fragmentation (Fig. 8C and D). However, this effect was lost upon treatment with edaravone, bringing the antibiotic-exposed cells to basal wild-type levels (Fig. 8C and D). These data further substantiate a critical role of both endogenously produced or fluoroquinolone-induced hydroxyl radicals on the kinetics of fluoroquinolone-mediated bactericidal activity on the pneumococcus, suggesting that modulation of redox exposure could result in fluoroquinolone tolerance.

### Tolerance via reduced redox stress promotes pneumococcal survival during antibiotic treatment of infection

**Fig 9:**
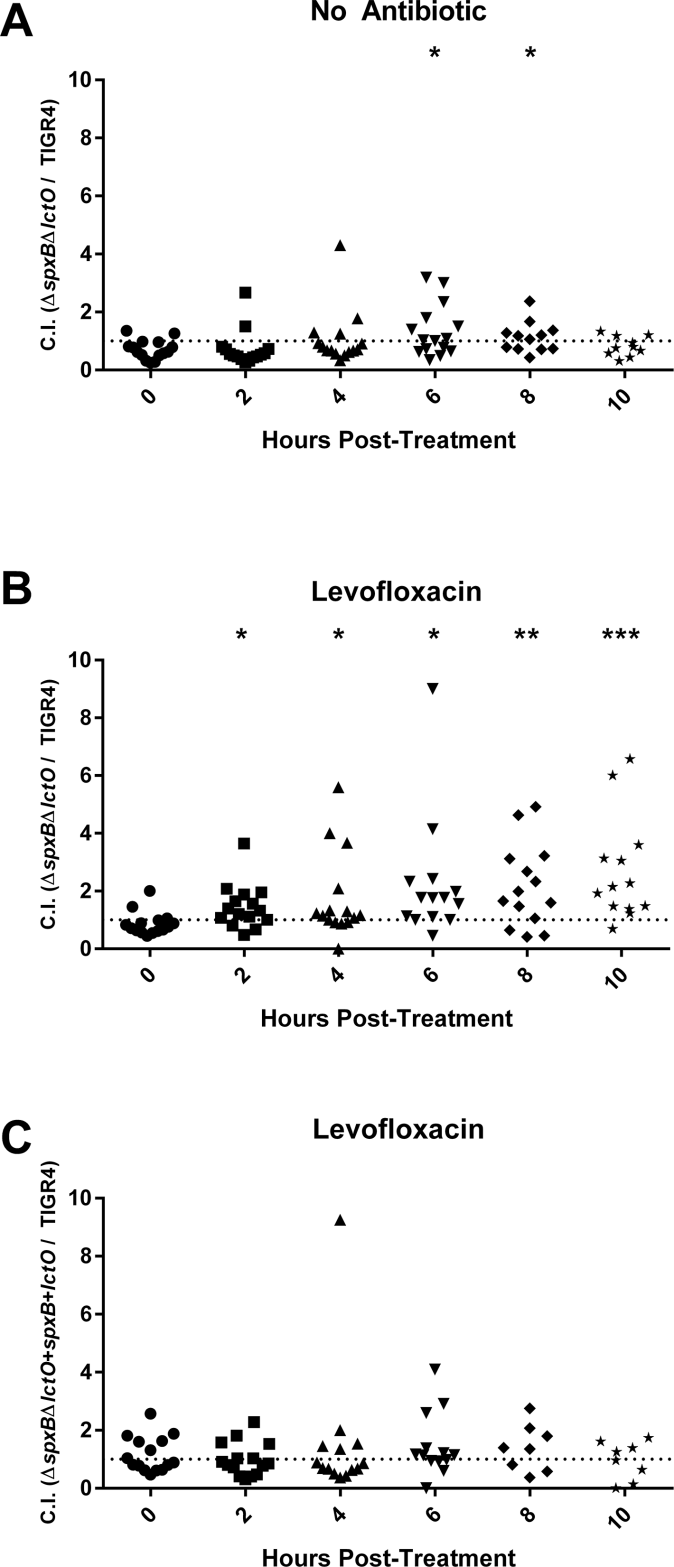
The *S. pneumoniae* strains lacking hydrogen peroxide production outcompetes wild type upon treatment with levofloxacin. Mice were infected with TIGR4 wild-type and the ∆*spxB*∆*lctO* double mutant (**A and B**) or TIGR4 and the complemented ∆*spxB*∆*lctO* double mutant (**C**) (*N* = 15). Mice were treated with PBS (**A**) or with 25 mg/kg levofloxacin (**B and C**) and bacterial burden in the blood was determined every 2 h post-treatment. Competitive indexes (CIs) of the ∆*spxB*∆*lctO* double mutant compared to TIGR4 (**A and B**) and of the complemented ∆*spxB*∆*lctO* double mutant compared to TIGR4 (**C**) were calculated. The CI of timepoints 2 through 10 h was compared to that of time point 0 for each condition using unpaired parametric *t* test in Prism 6. *P < 0.05, **P < 0.01, and ***P < 0.001. Each data point represents an individual mouse that was tracked consecutively over the respective time points. Dashed line represents a CI of 1.

These data suggest that mutations that reduce stress by oxygen radicals while retaining fitness may be one strategy whereby the pneumococci could evade the bactericidal activities of fluoroquinolones during infection. To test this, we measured fitness of the ∆*spxB*∆*lctO* double mutant in a competitive index challenge whereby the bacterial burden for individual mice was followed prior to and following treatment with levofloxacin. When levofloxacin was not administered (Fig. 9A), minimal differences in competitive index between the wild-type and double mutant were observed. Likewise, immediately prior to treatment, the competitive index was close to 1, which represents equal proportion of each strain (Fig. 9B). Starting at 2 h and continuing up to 10 h post-levofloxacin treatment, the ∆*spxB*∆*lctO* double mutant demonstrated a significant fitness advantage over the wild type (Fig. 9B). In contrast, no significant differences were observed up to 10 h post-levofloxacin treatment in a competition with TIGR4 and the complemented ∆*spxB*∆*lctO* double mutant (Fig. 9C). Taken together, these data suggest that metabolic solutions conferring antibiotic tolerance can lead to antibiotic treatment failure in the absence of traditional resistance mechanisms.

## DISCUSSION

In this study, we aimed to investigate the discrepancy between the high usage rate of fluoroquinolones and the low resistance rates observed in *S. pneumoniae* clinically. We found that single point mutations in either of the on-target enzymes, topoisomerase IV *parC* and DNA gyrase *gyrA*, conferred severe fitness costs for invasive disease unless a second point mutation was acquired simultaneously. This agrees with clinical studies reporting that high-level fluoroquinolone-resistant *S. pneumoniae* isolates often harbor double point mutations in *parC* and *gyrA* (52–54), consistent with our observations that double mutants exhibited high resistance while maintaining virulence (55). Rozen et al. also reported that double mutations in *parC* and *gyrA* not only increased resistance potential, but also lessened the *in vitro* fitness costs associated with fluoroquinolone resistance mutations (56). Our results do not preclude the presence of additional compensatory mechanisms, particularly for invasive clinical isolates for which only single mutations in either *parC* or *gyrA* have been identified (43, 57–59).

In agreement with the association of fitness costs with *de novo* generation of fluoroquinolone resistance, repeated exposure during *in vivo* evolution did not result in the emergence of mutations in either *gyrA* or *parC*. This agrees with previous studies in rabbit models whereby repeated exposures to either levofloxacin or moxifloxacin did not result in appreciable MIC shifts in *S. pneumoniae* recovered from infection (60). These data suggest that steep *in vivo* fitness tradeoffs constrain the emergence and spread of fluoroquinolone resistance in pneumococcus.

In this study, sequencing and functional characterization of the evolved populations indicated an important role of modulation of hydroxyl radical exposure for the observed tolerance phenotypes, a finding subsequently confirmed via targeted mutagenesis and chemical inhibition. The involvement of reactive oxygen species in antibiotic-mediated killing has been demonstrated by several studies, though the precise mechanisms at play and the extent of involvement of ROS remains debated (19, 61–63). Reactive oxygen species production has been shown to be a critical aspect of the post-antibiotic effect for delayed regrowth of fluoroquinolone treated *S. pneumoniae* (51). Ciprofloxacin, along with penicillin and kanamycin, have been demonstrated to increase the intracellular reactive oxygen species detected in *S. pneumoniae* (64). Moreover, fluoroquinolones generate oxidative stress through the Fenton reaction and, in response to exposure, genes involved in iron transport are downregulated (61, 65). In *E. coli*, the reduction of oxidative stress promotes survival upon exposure to bactericidal antibiotics (66). Oxidation of dCTP underlies antibiotic lethality mediated by reactive oxygen species in mycobacteria via induction of double-stranded breaks in genomic DNA (62), similar to our observations of decreased DNA fragmentation in the ∆*spxB*∆*lctO* double mutant following fluoroquinolone treatment. In contrast, increased reactive oxygen species levels in host cells infected by pneumococci can induce intracellular bacterial tolerant cells recalcitrant to fluoroquinolone-mediated killing (67). Induction of oxidative stress responses can result in a dramatic increase in the capacity of tolerant cells to survive treatment with fluoroquinolones via increased expression of multidrug-resistance pumps (68). Deletion of *spxB* renders pneumococci more susceptible to exogeneous hydrogen peroxide (69), which may enhance such host-mediated tolerance formation, and explain the evolutionary emergence of these mutations during successive *in vivo* passaging. Targeting NAD+ regeneration, critical for cellular redox balance, has also been demonstrated to enhance the bactericidal activity of antibiotics during invasive pneumococcal disease (70). These studies underscore the complex nature of the role of redox stress from both host and pathogen-derived sources for antibiotic efficacy.

Despite the importance of redox-active pathways for the activity of fluoroquinolone-mediated killing, robust bactericidal activity still was observed under anaerobic conditions in this study, indicating that distinct mechanisms dependent on the specific growth conditions are likely involved. One possibility is the disruption of cellular metabolic networks also plays an important role in the tolerance phenotypes, which is supported by previous Tn-seq screens whereby mutations in several metabolic pathways were found to confer fitness benefits under antibiotic pressure (71). As antibiotics can induce profound metabolic disruptions contributing to their bactericidal activity (72), mutations of metabolic gene networks to facilitate tolerance to antibiotics is a frequent mechanism adopted by bacterial pathogens in response to different classes of antibiotics. Metabolic profiling of cells exposed to bactericidal antibiotics reveal complex metabolic alterations in several pathways including nucleotide oxidation and protein carbonylation (72). Perturbation of fatty acid metabolic networks has been demonstrated to induce an antibiotic-tolerant state in pathogenic Mycobacterial species, suggesting important roles for carbon source utilization in antibiotic efficacy (73). Zhang et al. demonstrated that activated PpnN (YgdH) in *E. coli* participated in the inhibition of nucleotide synthesis, conferring tolerance to ciprofloxacin and ofloxacin via modulation of the stringent response (74). Mutations that enhance cytoplasmic acidification and subsequent shutdown of protein synthesis can promote the emergence of tolerant cell states (75). Additional studies have implicated that the buildup of toxic reactive metabolites under anaerobic conditions contribute to the bactericidal activity of antibiotics (76). These studies demonstrate that cellular metabolic processes and antibiotic activity are linked in multitude of ways (77) and suggest the potential for manipulation of the metabolic state of bacteria to potentiate antibiotic-mediated killing (78). Taken together, these studies underscore the complex interplay between cellular metabolism and antibiotic activity and indicate that the contribution of metabolic networks and both host and pathogen-derived redox stress may be important aspects for the capacity of the pathogen to evade antibiotic-mediated killing. We speculate that the tolerance phenotypes observed in this study are likely due to both the reductions in oxidant radical production as well as alterations in the cellular metabolic state.

Of note, the evolved isolates conferred tolerance to levofloxacin but not to ciprofloxacin, despite the loss of hydrogen peroxide production that was operative in tolerance to multiple fluoroquinolones. This may be due to additional mutations present in the mixed, complex population of the evolved isolates that tailor the tolerance phenotype specifically for levofloxacin at the expense of cross-protection against other drugs. The observation that scavenging intracellular oxidative molecules via edaravone treatment confers a tolerance phenotype while reversing the DNA fragmentation provides additional evidence that DNA fragmentation is an important aspect of the bactericidal activities of fluoroquinolones (66). These data also suggest that unforeseen activities of non-antibiotic drugs may have unintended consequences on bacterial physiology that reduce antibiotic efficacy. The data shown here further support the importance of redox stress for the bactericidal activity of fluoroquinolones and suggest that mutations in metabolic networks represent one pathway by which pathogens can evade antibiotic therapy.

Since metabolic adaptations that facilitate antibiotic tolerance may lead to antibiotic treatment failure and facilitate the acquisition of high-level resistance, targeting such pathways may prove a useful avenue for more effective treatment strategies. There is increasing recognition of the collateral consequences of antibiotic resistance acquisitions being a viable target for combination antibiotic therapy. Detailed analysis of the fitness costs of antibiotic resistance can provide insight into unique genetic and phenotypical liabilities that can be therapeutically targeted (79). Identification of highly conserved pathways that can be therapeutically targeted to potentiate the activity of antibiotics, particularly against tolerant populations, provides avenues to expand the therapeutic lifespan of current antibiotics (80). Our findings suggest metabolic pathways that increase cellular redox stress may prove to be attractive candidates for such approaches against *S. pneumoniae* infections.

## MATERIALS AND METHODS

### Media and growth conditions

*S. pneumoniae* was either grown in C+Y (a chemically defined semi-synthetic casein liquid medium supplemented with 0.5% yeast extract) pH 7.8, ThyB media (Todd Hewitt Broth + 0.2% yeast extract; BD), or on blood agar plates containing tryptic soy agar (EMD Chemicals, New Jersey), 3% defibrinated sheep blood, and 20 µg/mL neomycin. For all culture incubations, cells were incubated at 37°C, 5% CO_2_. For transformations, the respective fluoroquinolone for selection was added to the blood agar plates at concentrations indicated. Strains used in this study are indicated in Tables S2, S3, and S4. For growth curves, OD_620_ of cultures was monitored using Biotek Cytation 3 plate reader for 24 h. Growth curves of mutants were compared to that of TIGR4 via two-way ANOVA, repeated measures.

### MIC determination

To determine MIC of cells on agar plates, 100 µL of strains grown in ThyB to OD_620_ ~0.4 was plated and a levofloxacin, ciprofloxacin, or moxifloxacin E-test strip (bioMerieux) was added onto the center of each plate. MIC (µg/mL) was determined to be the concentration at which the symmetrical inhibition ellipse edge intersects the E-test strips on the plate (81). To determine MIC of cells in broth, titered frozen stocks were diluted 1:100 in C+Y with increasing concentrations of antibiotic (0–4 µg/mL for levofloxacin and 0–8 µg/mL for ciprofloxacin) in a 96-well plate. OD_620_ of cultures was monitored using Biotek Cytation 3 plate reader over 24 h.

### Genomic DNA extraction

Cells were grown in ThyB to OD_620_ ~0.6 and resuspended in genomic lysis buffer containing 10% DOC, 10% SDS, and 10 mg/mL proteinase K (Sigma). Cell lysates were subjected to standard phenol-chloroform extraction, followed by ethanol precipitation of gDNA (82).

### Transformation

*S. pneumoniae* was inoculated in C+Y and incubated at 37°C, 5% CO_2_ until OD_620_ ~0.07–0.1 at which time DNA and 3 µL of 1 mg/mL CSP1, CSP2, or both, were added to 1 mL of the bacterial cultures of D39, TIGR4, or CDC001, respectively. Three different types of DNA were used for transformation experiments: 2 kb PCR fragments encoding select point mutations, genomic DNA of fluoroquinolone resistant *S. pneumoniae*, or genomic DNA of *S. viridans* clinical isolates. Presence of resistance mutations was confirmed via whole-genome sequencing and MIC testing (Tables S2 and S3; Fig. S4). Following addition of DNA and CSP, cultures were incubated for 3 h, followed by plating of 100 µL of the transformation mixture onto five blood agar plates containing 2 µg/mL levofloxacin or 4 µg/mL ciprofloxacin. Colony-forming units (CFUs/mL) were enumerated at 24 and/or 48 h of incubation. Transformation efficiency was calculated as the ratio of transformant colonies (CFUs/mL) on select antibiotic plates to the total number of colonies (CFUs/mL) on plates without antibiotic. For all transformations, a Tn-seq library served as a positive control for competence and transformation efficiency with each donor DNA was compared to that of the TN-seq library via Mann-Whitney using Prism 6. For transformation of TIGR4 with PCR containing S81F *gyrA* point mutation, two different colony morphologies were observed: colonies producing reduced levels of capsule and colonies producing wild-type (normal) levels of capsule as determined by ELISA.

### Generation of complemented double mutant strain

The TIGR4 Δ*spxB*Δ*lctO* double mutant was complemented by introduction of both *spxB* and *lctO*, with their predicted native promoters and terminators, into the neutral CEP locus (83), along with a kanamycin resistance cassette for selection. *spxB* and its native promoter (including the CcpA binding region) and terminator were amplified from TIGR4 gDNA using primer pair (CTAGATTTCTTTGTTATAAAACAGAAATGACAG/CAAGAGTTTTGCTTAAAAACCATCTTACTCTCCTCCATAAAAAGACCGGATTG). *lctO* and its native promoter and terminator were amplified from TIGR4 gDNA using primer pair (GATGGTTTTTAAGCAAAACTCTTGAAAATGATTG/CTCTACTTATAAAACATTGTTAGAAATCGATTTG). The kanamycin resistance cassette (*km*^R^) was amplified from pABG5 plasmid (84) using primer pair (CTAACAATGTTTTATAAGTAGAGGATAAACCCAGCGAACCATTTGAGGTG/ATACAAATTCCTCGTAGGCGCTC). A *spxB-lctO-km*^R^ amplicon was generated through SOEing PCR (85) of the three fragments, whereby the 5’ sequences in the primers used to amplify *spxB* and *km*^R^ are complementary overhang sequences of the 5′ and 3′ ends of *lctO*, respectively. The upstream fragment for the CEP insertion was amplified using primer pair (GCAAATCTTTGGCTTCTTGTTCAAATTTTC/CTGTTTTATAACAAAGAAATCTAGCTACACAAAATAGGCTCCATAATATCCATAGGG) and the downstream fragment for the CEP insertion was amplified using primer pair (CTGTTTTATAACAAAGAAATCTAGCTACACAAAATAGGCTCCATAATATCCATAGGG/ATGTCTGAAAAATTAGTAGAAATCAAAGATTTAGAAATTTC). A CEPΩ*spxB-lctO-km*^R^ amplicon was generated through SOEing PCR of the CEP upstream and downstream and the *spxB-lctO*-kan^R^ amplicon, whereby the 5’ sequences in the primers used to amplify the CEP upstream and the CEP downstream are complementary overhang sequences of the 5′ end of *spxB* and 3′ ends of *km*^R^, respectively. The TIGR4 Δ*spxB*Δ*lctO* double mutant was transformed with the CEPΩ*spxB-lctO-km*^R^ amplicon following the protocol above and transformants were selected on plates containing 400 µg/mL of kanamycin.

### *In vitro* passaging

TIGR4 was diluted from frozen glycerol stocks 1:100 in ThyB containing increasing subinhibitory concentrations of either ciprofloxacin or levofloxacin and incubated until reaching OD_620_ ~0.6 after which the culture was mixed with glycerol at a final concentration of 20% and stocks were stored at −80°C before serving as an inoculum for the next passage. Each subsequent passage contained progressively increasing twofold concentration of the respective antibiotic, ranging from 0.125 to 1 µg/mL for levofloxacin and from 0.25 µg/mL to 8 µg/mL for ciprofloxacin for 30 passages.

### Whole-genome sequencing

Genomic sequence libraries were prepared using Nextera kits and sequenced accordingly on Illumina HiSeq. All sequence reads are available at NCBI under accession # PRJNA407467. The reads were compared to the TIGR4 reference genome (AE005672.3) using breseq for identification of mutations resulting from recombination events and *de novo* point mutations (86).

### Genotype inferences from populations and Muller plots

Mutation filtering, allele frequencies, and plotting were done in R v3.5.3. Muller plots were generated using the lolipop package (https://github.com/cdeitrick/lolipop) v0.6 using default parameters. To summarize, these tools predict genotypes and lineages based on shared trajectories of mutations over time and test their probability of nonrandom genetic linkage. Successive evolution of genotypes, or nested linkage, is identified by a hierarchical clustering method. The method also includes customizable filters that eliminate singletons that do not comprise prevalent genotypes. Muller plots were color-coded by the presence of putative driver mutations within each genotype.

### Antibiotic kill curves

The wild-type TIGR4, mutants generated in TIGR4, and the experimentally evolved isolates were inoculated in ThyB at OD_620_ ~0.05. When cells reached OD_620_ = 0.2, the culture was split into untreated and treated conditions. For treatment with antibiotics, the bacteria were exposed to 4 µg/mL levofloxacin, 8 µg/mL ciprofloxacin, or 0.48 µg/mL moxifloxacin. These concentrations are equivalent to 4× MIC determined by µbroth testing and provide the highest stringency without complete clearance within the timeframe of the experiment. For treatment with edaravone, 3-methyl-1-phenyl-2-pyrazolin-5-one (Edaravone; Millipore 443300) was resuspended in ethanol at 500 mM and heated at 65°C for solubilization. Edaravone was added to the culture at a final concentration of 1 mM concurrently with the antibiotics. After addition of treatment, the cultures were incubated for 4 h. For anaerobic conditions, when cells reached OD_620_ = 0.2, the culture was pelleted for 5 min, 6,000 × *g*. Keeping the cellular pellet on ice, the media were deoxygenated by incubation with oxyrase (Oxyrase) at 10% culture volume at 37°C, 5% CO_2_ for 30 min. The cellular pellet was then resuspended in the deoxygenated media, followed by treatment with antibiotics. For all conditions, bacterial cell number was determined hourly by serial dilution of the culture and plating for CFU enumeration. Cell numbers were plotted as log_10_ (CFU/mL) and the log_10_ (CFU/mL) of TIGR4 was compared to that of each lineage or mutant using two-way ANOVA multiple comparisons, comparing the mean of each time point in Prism 6.

### TUNEL assay

TIGR4 was inoculated into ThyB at OD_620_ ~0.05 from TSA blood agar plates. When cells reached OD_620_ = 0.2, the culture was split into four 25 mL aliquots plus one 5 mL aliquot. The 5 mL aliquot represents the 0-h treatment. Each 25 mL aliquot was treated with no antibiotic or 4 µg/mL levofloxacin or 8 µg/mL ciprofloxacin and either no edaravone or 1 mM edaravone. After treatment, the cultures were incubated for 4 h. Every hour, 5 mL of culture was transferred from the 25 mL culture to a new conical tube. About 100 µL was used to determine bacterial cell number via serial dilution of the culture and plating. Each 5 mL of culture was centrifuged 6,000 × *g*, 5 min and supernatant was removed. To measure the level of DNA fragmentation via TUNEL staining, the cellular pellet was fixed and stained using a TUNEL kit (BD #556381) via adaptation of the manufacturer’s protocol. At each time point, the cellular pellet was fixed in 1 mL of 1% paraformaldehyde for 1 h on ice. The pellet was washed with 1× phosphate-buffered saline (PBS) three times and resuspended in 70% ethanol, followed by incubation at −20°C overnight. The pellet was stained according to the manufacturer’s protocol, with a final resuspension of 300 µL. Levels of FITC (535/623) and PI (488/520) were detected by transferring 200 µL of the resuspended pellet into a black-bottom 96-well plate and measuring fluorescence on a BioTek Cytation 3. DNA damage was calculated as the level of FITC (apoptotic cells) divided by the level of PI (total DNA). DNA damage over time was calculated by division of DNA damage at time point 1 through 4 h by the DNA damage at time point 0. Additional controls for the TUNEL assay include the correlation between PI values and CFU/mL as a measure of cell density and results obtained from the positive and negative controls (Fig. S7). Zero millimolar edaravone, 0 antibiotic was compared to that of 1 mM edaravone, 0 antibiotic using two-way ANOVA multiple comparisons, comparing the mean of each timepoint in Prism 6. Zero millimolar edaravone, 0 antibiotic was compared to that of 0 mM edaravone, with antibiotic and 1 mM edaravone, 0 antibiotic was compared to that of 1 mM edaravone, with antibiotic similarly.

### H_2_O_2_ production measurement

Hydrogen peroxide production was measured via two independent methods. In the first, TIGR4 and the evolved isolates were grown in ThyB until OD_620_ ~0.4, centrifuged, and resuspended in PBS. After incubation in PBS for 30 min, the bacteria were pelleted via centrifugation. The supernatant was collected for the Amplex Red hydrogen peroxide/peroxidase assay (ThermoFisher Scientific #A22188) and 50 µL Amplex Red reagent was added to 50 µL of the supernatant in each microplate well. The absorbance value (OD_562_) was measured using a BioTek Cytation 3 plate reader. Levels of H_2_O_2_ of each strain were calculated as µmoles of hydrogen peroxide per mg of cellular protein**,** determined via a bicinchoninic acid (BCA) assay (Pierce). H_2_O_2_ levels were compared with unpaired parametric *t* test in Prism 6. In the second method, serial dilutions of cultures were plated on ThyB plates with horseradish peroxidase (0.2 mg/mL) and 2,2′-azinobis(3-ethylbenzothiazoline-6-sulphonic acid) (3 mg/mL). Plates were incubated in a GasPak (BD) anaerobic chamber overnight at 37°C and H_2_O_2_ production was observed by colorimetric changes in the colonies upon exposure to ambient air.

### Bacterial ELISA

Whole-cell bacterial ELISA was performed as a quantitative measure of capsule on the bacterial surface. TIGR4 and the S81F *gyrA* mutant variants were grown to OD_620_ ~0.4 in C+Y, pelleted, and diluted 1:5 in coating buffer (sodium carbonate). Bacteria were bound to 96-well plates via centrifugation. Plates were dried and subjected to blocking buffer (10% FBS). Bound cells were probed with type-4 capsular antibody (Statens Serum Institute) and LytA antibody, followed by probing with AP-conjugated secondary antibody (Southern Biotech) and detection using an AP yellow ELISA substrate (Sigma). Capsule immunoreactivity was normalized to immunoreactivity to LytA.

### *In vivo* virulence

To determine virulence *in vivo*, 6-week-old female BALB/c mice (Jackson Laboratory) were intranasally infected with the wild-type TIGR4 or mutant strains (10^6^ CFUs in 100 µL). At 24 and 48 h post-challenge, 2 µL of nasal samples and 5 µL of blood samples were collected via nasal lavage and tail bleeds for enumeration of bacterial burden. Mice were monitored for disease progression for 10 days. Bacterial titers (CFUs/mL) were compared using non-parametric Mann-Whitney *t* test and survival data were analyzed with Mantel-Cox log rank tests in Prism 6.

### *In vivo* passaging

To model the evolution of fluoroquinolone resistance *in vivo*, three groups of 7-week-old female BALB/c mice (Jackson Laboratory) were intranasally infected with 100 µL containing 10^6^ CFUs of TIGR4. This inoculum was chosen to permit a large enough population to be recovered for downstream applications while ensuring mouse survival. Six hours post-infection, mice were administered levofloxacin intraperitoneally. The initial dosage of levofloxacin (25 mg/kg) was utilized as it eliminated 90–99% of the bacterial population (Fig. S8). After 15 passages, the levofloxacin dosage was increased in a stepwise fashion to increase antibiotic selective pressure. For each passage, 12 h after levofloxacin treatment, mice were euthanized, the lungs were homogenized, and bacteria in lung homogenates were plated for subsequent collection for frozen aliquots. Frozen aliquots from each passage served as the inoculum to infect the next group of mice for the subsequent, respective lineage, and the remaining aliquots were used for gDNA extraction of the recovered bacterial population. Overall change in bacterial burden across passaging was compared via Krukal-Wallis one-way ANOVA and bacterial burden in passages 2 through 30 was compared to that of passage 1 via Mann-Whitney using Prism 6.

### *In vivo* kill kinetics

To model the impact of antibiotic tolerance *in vivo*, 8-week-old female BALB/c mice (Jackson Laboratory) were intraperitoneally infected with either the wild-type TIGR4 and the Δ*spxB*Δ*lctO* mutant strain concurrently (5 × 10^5^ CFUs of each strain in 100 µL) or the wild-type TIGR4 and the complemented Δ*spxB*Δ*lctO* mutant strain concurrently (5 × 10^5^ CFUs of each strain in 100 µL). Sixteen hours post-infection, a blood sample was taken via tail bleeding (time point 0). Mice were administered a dose of levofloxacin at 25 mg/kg (*N* = 15) intraperitoneally. As a control, 8-week-old female BALB/c mice (Jackson Laboratory) were intraperitoneally infected with the wild-type TIGR4 and the Δ*spxB*Δ*lctO* mutant strain concurrently (5 × 10^4^ CFUs of each strain in 100 µL). Sixteen hours post-infection, a blood sample was taken via tail bleeding (timepoint 0) and mice were administered a dose of 1×PBS (*N* = 15) intraperitoneally. For all mice, blood samples were taken every 2 h via tail bleeding. Bacterial burden in the blood was determined by serial dilution of the blood and spotting on duplicate plates with or without 1 µg/mL erythromycin to differentiate wild type versus mutant. The CFU/mL of the Δ*spxB*Δ*lctO* double mutant and the complement strain was the CFU/mL on plates with erythromycin. The CFU/mL of TIGR4 was calculated as the CFU/mL on the plates without erythromycin (total bacteria) minus the CFU/mL on the plates with erythromycin (Fig. S9). The competitive index (CI) in Fig. 9 was calculated by dividing the CFU/mL of the Δ*spxB*Δ*lctO* double mutant or the complement strain by the CFU/mL of TIGR4. The CI of time points 2 through 10 were compared to the competitive index of time point 0 using unpaired parametric *t* test with Prism 6. To monitor the changes in bacterial burden of each strain over time, the CFU/mL in the blood of each mouse was plotted over time (Fig. S10; different colored lines represent individual mice). Similarly, the CI at each time point was plotted for each mouse (Fig. S10C, H and I).

## Data Availability

All data are available in the main text or the supplemental materials.

## SUPPLEMENTAL MATERIAL

Supplemental Material mbio.02828-23-s0001.docx Supplemental tables and figures.

ASM does not own the copyrights to Supplemental Material that may be linked to, or accessed through, an article. The authors have granted ASM a non-exclusive, world-wide license to publish the Supplemental Material files. Please contact the corresponding author directly for reuse.
