# Supplementary material for: Streptococcus pneumoniae favors tolerance via metabolic adaptation over resistance to circumvent fluoroquinolones
Source: mBio. 2024 Jan 9;15(2):e02828-23. doi: 10.1128/mbio.02828-23 (PMC10865975; doi:10.1128/mbio.02828-23)
Supplement: Supplemental Material — Supplemental tables and figures. [file mbio.02828-23-s0001.docx]

**Supplementary Material**

Supplemental Table 1. Prevalence of fluoroquinolone resistance from 1988 to 2015

| **Fluoroquinolone Usage (Prescriptions per 1000 Persons)** | **Fluoroquinolone Prevalence** | **Periods** | **Region** | **Ref** |
| --- | --- | --- | --- | --- |
| 37.5 | 1.2% isolates first step mutation in *parC*  6.7% efflux phenotype | 2002-2006 | Germany | ^1^ |
| Not available | 0.2% resistant to levofloxacin | 2004-2005 to 2014-2015 | Germany | ^2^ |
| Ciprofloxacin  38.2 to 59.4 | 1.8% resistant to ciprofloxacin | 1988-2006 | Canada | ^3-5^ |
| Respiratory fluoroquinolone 5.3 to 27.4 | 1.2% non-susceptible to levofloxacin 1.1% non-susceptible to moxifloxacin | 1988-2006 | Canada | ^3-5^ |
| Not available | 0% in 1993 to 1.7% in 1997 and 1998 | 1988 to 1997 | Canada | ^6^ |
| Not available | 1.5% 2.9% | 1993 to 1994  1997 to 1998 | Canada | ^1^ |
| Not available | <2% resistant to levofloxacin | 2007-2016 | Canada | ^4,7^ |
| 39 to 106 | < 2% resistant to levofloxacin  < 1% resistant to moxifloxacin | 1995-2002  2008 | USA | ^8,9^ |
| Not available | 4-13% resistant to fluoroquinolones (ciprofloxacin) | 2000 | Croatia and Hong Kong | ^10^ |
| Not available | 4% resistant to newer fluoroquinolones (levofloxacin, moxifloxacin) | 2009 to 2010 | Asia | ^11^ |
| Not available | 7.1% | 1998-1999 | Spain | ^12^ |
| Not available | 1.64 % resistant to ciprofloxacin, 0% to other fluoroquinolones | 2000-2002 | Hungary | ^13^ |

Supplemental Table 2. Strains used in this study and respective MICs as determined by E-test

| **Strains** | **Description** | **MIC (µg/mL)** | |  |
| --- | --- | --- | --- | --- |
|  |  | **Ciprofloxacin** | **Levofloxacin** | **Source** |
| TIGR4 | TIGR4 wild-type, serotype 4 | 4 | 1 | [www.tigr.org](http://www.tigr.org/) |
| CDC001 | Serotype 9V | 4 | 1 | CDC |
|  |  |  |  |  |
| D39 | D39 wild-type, serotype 2 | 4 | 1 |  |
| Tn-seq mutant library | TIGR4 | 4 | 1 | This study |
| S79Y *parC* | Single point mutation S79Y in topoisomerase IV *parC* | 8 | 2 | This study |
| S81F *gyrA* | Single point mutation S81F in gyrAse A *gyrA* | 8 | 2 | This study |
| S79Y *parC* & S81F *gyrA* | Double point mutations S79Y in *parC* and S81F in *gyrA* | 16 | 4 | This study |
| S79Y *parC* & D435N *gyrB* | Double point mutations S79Y in *parC* and D435N in *gyrB* | 8 | 2 | This study |
| 188 | Single point mutation S79Y in *parC*, serotype 11A |  | 4 | Nationales Referenzzentrum für Streptokokken  Abteilung Medizinische Mikrobiologie, Universitätsklinikum RWTH Aachen, Germany |
| 68 | Single point mutation in S81F in *gyrA*, serotype 11A |  | 4 | Nationales Referenzzentrum für Streptokokken  Abteilung Medizinische Mikrobiologie, Universitätsklinikum RWTH Aachen, Germany  Pauwelsstraße 30 |
| 66 | Double point mutations S79Y in *parC* and S81F in *gyrA*, serotype 9V |  | ≥32 | Nationales Referenzzentrum für Streptokokken  Abteilung Medizinische Mikrobiologie, Universitätsklinikum RWTH Aachen, Germany |
| 93 | Double point mutations S79Y in *parC* and S81F in *gyrA*, serotype 19F |  | ≥32 | Nationales Referenzzentrum für Streptokokken  Abteilung Medizinische Mikrobiologie, Universitätsklinikum RWTH Aachen, Germany |
| 112 | Double point mutations S79Y in *parC* and S81F in *gyrA*, serotype 7F |  | ≥32 | Nationales Referenzzentrum für Streptokokken  Abteilung Medizinische Mikrobiologie, Universitätsklinikum RWTH Aachen, Germany |
| 114 | Double point mutations S79F in *parC* and E435D in *gyrB*, serotype 19A |  | 8 | Nationales Referenzzentrum für Streptokokken  Abteilung Medizinische Mikrobiologie, Universitätsklinikum RWTH Aachen, Germany |
| 176 | Double point mutations S79F in *parC* and E474K in *gyrB*, serotype 11A |  | 6 | Nationales Referenzzentrum für Streptokokken  Abteilung Medizinische Mikrobiologie, Universitätsklinikum RWTH Aachen, Germany |
| 182 | Double point mutations S79F in *parC* and E435D in *gyrB*, serotype 23F |  | 24 | Nationales Referenzzentrum für Streptokokken  Abteilung Medizinische Mikrobiologie, Universitätsklinikum RWTH Aachen, Germany |
|  |  |  |  |  |
| TIGR4 Lineage 1 | *in vivo* experimentally evolved under levofloxacin pressure |  | 2 | This study |
| TIGR4 Lineage 2 | *in vivo* experimentally evolved under levofloxacin pressure |  | 2 | This study |
| TIGR4 Lineage 3 | *in vivo* experimentally evolved under levofloxacin pressure |  | 2 | This study |

Supplemental Table 3. Strains used in this study and respective MICs as determined by µBroth

| **Strains** | **Description** | **MIC (µg/mL)** | |  |
| --- | --- | --- | --- | --- |
|  |  | **Ciprofloxacin** | **Levofloxacin** | **Source** |
| TIGR4 | TIGR4 wild-type, serotype 4 | 2 | 1 | [www.tigr.org](http://www.tigr.org/) |
| TIGR4 Lineage 1 | *in vivo* experimentally evolved under levofloxacin pressure | 2 | 1 | This study |
| TIGR4 Lineage 2 | *in vivo* experimentally evolved under levofloxacin pressure | 2 | 1 | This study |
| TIGR4 Lineage 3 | *in vivo* experimentally evolved under levofloxacin pressure | 2 | 1 | This study |
| ∆*spxB*∆*lctO* | Double knockout in *spxB* and *lctO*  *Erm^R^, Spec^R^* | 2 | 1 | Echlin, et al. ^14^ |
| ∆*spxB*∆*lctO* +*spxB*+*lctO* | Double knockout in *spxB* and *lctO* complemented with *spxB* and *lctO*  *Erm^R^, Spec^R^, Kan^R^* | 2 | 1 | This study |
| S79Y *parC* | Single point mutation S79Y in topoisomerase IV *parC* | 8 | 2 | This study |
| S81F *gyrA*,  reduced capsule | Single point mutation S81F in gyrAse A *gyrA* | 8 | 4 | This study |
| S81F *gyrA*,  normal capsule | Single point mutation S81F in gyrAse A *gyrA* | 8 | 4 | This study |

Supplemental Table 4. *S. viridans* strains used in this study and respective MICs

| Strain | Description |  | Levofloxacin MIC (µg/mL) | Source |
| --- | --- | --- | --- | --- |
| 1 | Double point mutations S79Y in *parC* and S81F in *gyrA* |  | 64 | SJCRH Clinical Microbiology Laboratory |
| 2 | Double point mutations S79Y in *parC* and S81F in *gyrA* |  | 16 | SJCRH Clinical Microbiology Laboratory |
| 3 | Double point mutations S79Y in *parC* and S81F in *gyrA* |  | 64 | SJCRH Clinical Microbiology Laboratory |
| 5 | Triple mutations S79Y in *parC,* S81F in *gyrA,* and D474E in *gyrB* |  | 16 | SJCRH Clinical Microbiology Laboratory |
| 6 | Double point mutations S79Y in *parC* and S81F in *gyrA* |  | 16 | SJCRH Clinical Microbiology Laboratory |
| 7 | Double point mutations S79Y in *parC* and S81F in *gyrA* |  | 16 | SJCRH Clinical Microbiology Laboratory |
| 10 | Double point mutations S79Y in *parC* and S81F in *gyrA* |  | 64 | SJCRH Clinical Microbiology Laboratory |
| 12 | Double point mutations S79Y in *parC* and S81F in *gyrA* |  | 16 | SJCRH Clinical Microbiology Laboratory |
| 13 | Double point mutations S79Y in *parC* and S81F in *gyrA* |  | 32 | SJCRH Clinical Microbiology Laboratory |
| 15 | Double point mutations S79Y in *parC* and S81F in *gyrA* |  | 16 | SJCRH Clinical Microbiology Laboratory |
| 16 | Double point mutations S79Y in *parC* and S81F in *gyrA* |  | 64 | SJCRH Clinical Microbiology Laboratory |
| 17 | Double point mutations S79Y in *parC* and S81F in *gyrA* |  | 16 | SJCRH Clinical Microbiology Laboratory |
| 18 | Double point mutations S79Y in *parC* and S81F in *gyrA* |  | 16 | SJCRH Clinical Microbiology Laboratory |
| 20 | Double point mutations S79Y in *parC* and S81F in *gyrA* |  | 16 | SJCRH Clinical Microbiology Laboratory |





**Supplemental Figure 1. Growth kinetics of experimentally evolved isolates.** Strains were grown *in vitro* in semi-chemically defined media without antibiotics (A), in sub-MIC concentrations of antibiotics, including 0.5 µg/mL levofloxacin (B), 1 µg/mL ciprofloxacin (C), and 0.1 µg/mL moxifloxacin (D), and in higher concentrations of antibiotics, including 1 µg/mL levofloxacin (E), 2 µg/mL of ciprofloxacin (F), and 0.18 µg/mL of moxifloxacin (G). Strains included wild-type parental TIGR4 and three experimentally evolved lineages passaged under levofloxacin *in vivo*. Data represent at least three biological replicates with the mean and SEM plotted.





Supplemental Figure 2. *S. pneumoniae* demonstrated *de novo* resistance *in vitro*

passaging. *S. pneumoniae* was exposed to gradually increasing concentrations of both A) levofloxacin and B) ciprofloxacin for 30 days.


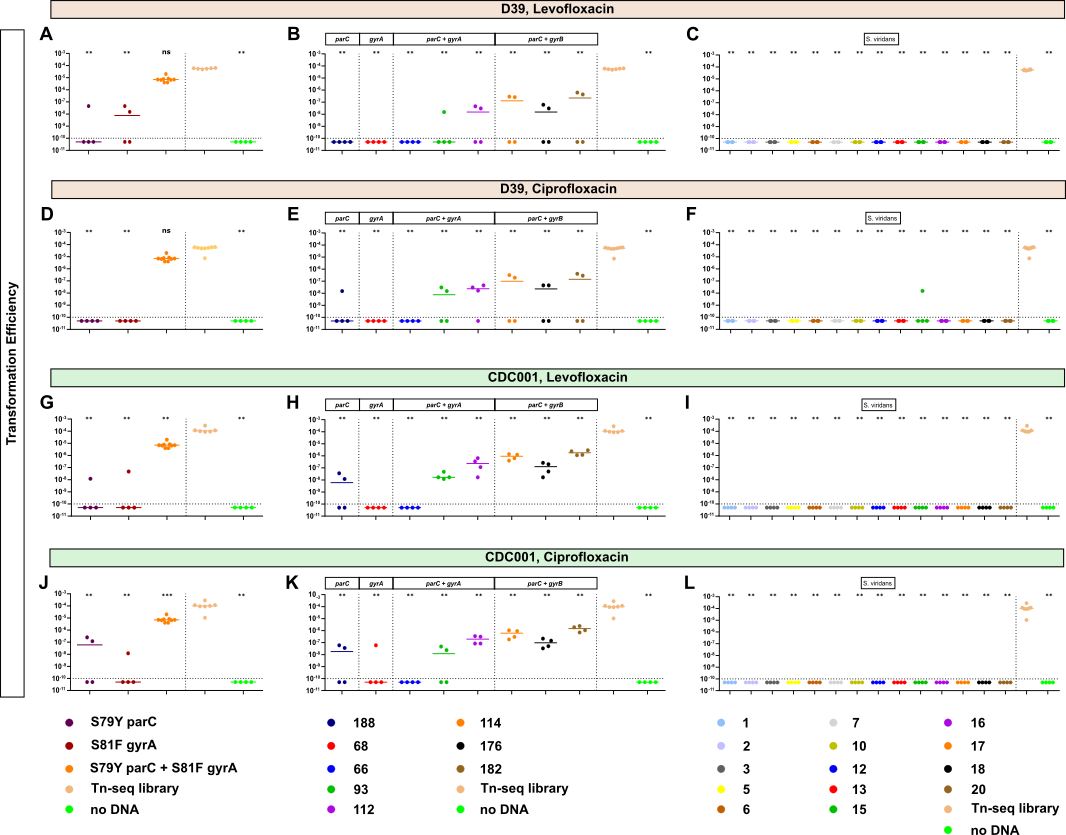


**Supplemental Figure 3. Transformation efficiency for fluoroquinolone resistance determinants across various strains.** Transformation efficiency of fluoroquinolone resistance determinants across multiple strain backgrounds including D39 (A-F) and CDC001 (G-L), serotypes 2 and 9V. Resistant colonies were recovered on plates supplemented with either 2 µg/mL levofloxacin (A-C and G-I) or 4 µg/mL ciprofloxacin (D-F and J-L). DNA used for transformation included either PCR fragments encoding the respective mutations (A, D, G, J), genomic DNA of *S. pneumoniae* clinical isolates harboring the respective mutations (B, E, H, K), or genomic DNA of *S. viridans* clinical isolates harboring fluoroquinolone resistance (C, F, I, L). Transformation efficiency was calculated as the ratio of the number of transformants (CFUs/mL) selected on either levofloxacin or ciprofloxacin to the number of total bacteria (CFUs/mL). For all transformations, a Tn-seq library served as a positive control for competence and transformation efficiency with each donor DNA was compared to that with the TN-seq library via Mann Whitney using Prism 6. **p<0.01, ***p<0.001. Each datapoint represents an individual biological replicate and bars represent median.





**Supplemental Figure 4. MIC of strains used in this study as determined by µbroth growth curve.** Strains were grown *in vitro* in semi-chemically defined media in increasing concentration of levofloxacin (A-G), ranging from 0 µg/mL to 4 µg/mL, or ciprofloxacin (H-N), ranging from 0 µg/mL to 8 µg/mL. Strains included the wild-type TIGR4 parental strain (A, E, H, and L), the S81F *gyrA* mutant with reduced levels of capsule (B, I), the S81F *gyrA* mutant with normal levels of capsule (C, J), the S79Y *parC* mutant (D, K), the Δ*spxB*Δ*lctO* mutant (F, M), and the complemented Δ*spxB*Δ*lctO* double mutant (G, N). Data represent three biological replicates with the mean and SEM plotted (A-D and H-K) and represent at least four biological replicates with the mean and SEM plotted (E-G and L-N).

**

**

**Supplemental Figure 5. Individual fluoroquinolone resistance mutations confer high fitness tradeoffs during invasive infection.** Two variants of TIGR4 S81F *gyrA* were identified and capsule production was measured by ELISA, with two biological replicates (A). To determine *in vitro* fitness of mutants, wild-type TIGR4 and the S81F *gyrA* mutant with reduced levels of capsule, the S81F *gyrA* mutant with normal levels of capsule, and the S79Y *parC* mutant were grown in semi-chemically defined media (B). Data represent eight biological replicates with the mean and SEM plotted. To determine *in vivo* fitness of mutants, mice were infected intranasally with the parental TIGR4 wild-type or the isogenic fluoroquinolone mutants. Nasal lavage at 24 hours (C) and 48 hours (D) post-challenge was used to ascertain relative bacterial colonization burden. Invasive potential was assayed via blood titers at 24 hours (E) and 48 hours (F) post-challenge. Infected mice were followed for ten days for relative survival between the wild-type and mutants (G). The bacterial burden data from all strains were compared to that of the wild-type TIGR4 via Mann-Whitney in Prism 6. Bars represent median, with each datapoint representing an individual mouse (N=5). *p<0.05, **p<0.01. Survival data were analyzed with Mantel-Cox log rank tests, with all mutant strains compared to wild-type TIGR4 having p <0.0001. Lines were shifted for visibility. Dashed line represents limit of detection.





Supplemental Figure 6. Complementation of double mutant restores H_2_O_2_ production. H_2_O_2_ production was measured using the Amplex Red kit. Strains included the wild-type TIGR4, the Δ*spxB*Δ*lctO* double knockout mutant, and the complemented double mutant. Levels were reported as µmoles of hydrogen peroxide per mg of cellular protein, determined via BCA assay. Strains were compared via unpaired parametric t-test in Prism 6. **p-value<0.01, ****p<0.0001. Data represent four biological replicates plotted as mean and SD.

**

Supplemental Figure 7.** **Controls for TUNEL assay.** Correlation between PI values and CFU/mL of 5 mL cultures used for TUNEL staining (A). Each datapoint represents CFU/ml and PI from strains in all conditions in Figure 8. Correlation was compared using two-tailed spearman with a p<0.0001. Ratio of FITC to PI levels of the positive and negative controls provided by the kit, detected at the same time as *S. pneumoniae* fixed cells (B). Bars represent mean and SD.

**

**

**Supplemental Figure 8. Levofloxacin dosing of infected mice.** Mice were intranasally infected with TIGR4 and, after eight hours of infection, mice were treated with 25 mg/ kg of levofloxacin. Immediately prior to treatment and 2-, 4-, and 8-hours post treatment, lungs from three mice for each timepoint were harvested and the bacterial burden was enumerated. Each datapoint represents an individual mouse and bars represent median.

**

**

**Supplemental Figure 9. Total CFU/mL used to calculate the competitive index in Figure 9.** Mice were infected with TIGR4 wild-type and the ∆*spxB*∆*lctO* double mutant (A-D) or TIGR4 and the complemented ∆*spxB*∆*lctO* double mutant (E, F) (N=15). Mice were treated with PBS (A, B) or with 25 mg/kg levofloxacin (C-F). Bacterial burden in the blood was determined every two hours post-treatment by plating on TSA agar plates with and without 1 µg/mL erythromycin. The CFU/mL of the Δ*spxB*Δ*lctO* double mutant (A, C) and the complemented double mutant (E) was the CFU/mL on plates with erythromycin. The CFU/mL of TIGR4 (B, D, F) was calculated as the CFU/mL on the plates without erythromycin (total bacteria) minus the CFU/mL on the plates with erythromycin. The C.I. in Figure 9A was calculated by dividing the CFU/mL of Δ*spxB*Δ*lctO* (A) by the CFU/mL of TIGR4 (B) for each mouse treated with no antibiotic. The C.I. in Figure 9B was calculated by dividing the CFU/mL of Δ*spxB*Δ*lctO* (C) by the CFU/mL of TIGR4 (D) for each mouse treated with 25 mg/kg levofloxacin. The C.I. in Figure 9C was calculated by dividing the CFU/mL of the complemented Δ*spxB*Δ*lctO* (E) by the CFU/mL of TIGR4 (F) for each mouse treated with 25 mg/kg levofloxacin. Each datapoint represents an individual mouse and bars represent median. The thick dashed line represents the inoculum titer for the infection and the thin dashed line represents the limit of detection (A-F).

**

**

**Supplemental Figure 10. Total CFU/mL and competitive index in each mouse followed over 10 hours post treatment.** Mice were infected with TIGR4 wild-type and the ∆*spxB*∆*lctO* double mutant (A-F) or TIGR4 and the complemented ∆*spxB*∆*lctO* double mutant (G-I) (N=15). Mice were treated with PBS (A-C) or with 25 mg/kg levofloxacin (D-I). Bacterial burden in the blood was determined every two hours post-treatment by plating on TSA agar plates with and without 1 µg/mL erythromycin. The CFU/mL of the Δ*spxB*Δ*lctO* double mutant (A, D) and the complemented double mutant (G) was the CFU/mL on plates with erythromycin. The CFU/mL of TIGR4 (B, E, H) was calculated as the CFU/mL on the plates without erythromycin (total bacteria) minus the CFU/mL on the plates with erythromycin. The C.I. in mice treated with no antibiotic (C) was calculated by dividing the CFU/mL of Δ*spxB*Δ*lctO* (A) by the CFU/mL of TIGR4 (B). The C.I. in mice treated with 25 mg/kg levofloxacin (F, I) was calculated by dividing the CFU/mL of Δ*spxB*Δ*lctO* (D) by the CFU/mL of TIGR4 (E) or by dividing the CFU/mL of the complemented Δ*spxB*Δ*lctO* (G) by the CFU/mL of TIGR4 (H). The thick dashed line represents the inoculum titer for the infection and the thin dashed line represents the limit of detection (A, B, D, E, G, H). Dashed line represents a C.I. of 1 (C, F, I). Different colored lines represent individual mice.

1 Pletz, M. W. *et al.* Low prevalence of fluoroquinolone resistant strains and resistance precursor strains in Streptococcus pneumoniae from patients with community-acquired pneumonia despite high fluoroquinolone usage. *Int J Med Microbiol* **301**, 53-57, doi:10.1016/j.ijmm.2010.05.004 (2011).

2 Schmitz, J. *et al.* Fluoroquinolone resistance in Streptococcus pneumoniae isolates in Germany from 2004-2005 to 2014-2015. *Int J Med Microbiol* **307**, 216-222, doi:10.1016/j.ijmm.2017.04.003 (2017).

3 Adam, H. J., Hoban, D. J., Gin, A. S. & Zhanel, G. G. Association between fluoroquinolone usage and a dramatic rise in ciprofloxacin-resistant Streptococcus pneumoniae in Canada, 1997-2006. *Int J Antimicrob Agents* **34**, 82-85, doi:10.1016/j.ijantimicag.2009.02.002 (2009).

4 Patel, S. N. *et al.* Susceptibility of Streptococcus pneumoniae to fluoroquinolones in Canada. *Antimicrob Agents Chemother* **55**, 3703-3708, doi:10.1128/AAC.00237-11 (2011).

5 Patel, S. N., Melano, R., McGeer, A., Green, K. & Low, D. E. Characterization of the quinolone resistant determining regions in clinical isolates of pneumococci collected in Canada. *Ann Clin Microbiol Antimicrob* **9**, 3, doi:10.1186/1476-0711-9-3 (2010).

6 Chen, D. K., McGeer, A., de Azavedo, J. C. & Low, D. E. Decreased susceptibility of Streptococcus pneumoniae to fluoroquinolones in Canada. Canadian Bacterial Surveillance Network. *N Engl J Med* **341**, 233-239, doi:10.1056/NEJM199907223410403 (1999).

7 Golden, A. R. *et al.* Comparison of antimicrobial resistance patterns in Streptococcus pneumoniae from respiratory and blood cultures in Canadian hospitals from 2007-16. *J Antimicrob Chemother* **74**, iv39-iv47, doi:10.1093/jac/dkz286 (2019).

8 Linder, J. A., Huang, E. S., Steinman, M. A., Gonzales, R. & Stafford, R. S. Fluoroquinolone prescribing in the United States: 1995 to 2002. *Am J Med* **118**, 259-268, doi:10.1016/j.amjmed.2004.09.015 (2005).

9 Jones, R. N., Sader, H. S., Moet, G. J. & Farrell, D. J. Declining antimicrobial susceptibility of Streptococcus pneumoniae in the United States: report from the SENTRY Antimicrobial Surveillance Program (1998-2009). *Diagn Microbiol Infect Dis* **68**, 334-336, doi:10.1016/j.diagmicrobio.2010.08.024 (2010).

10 Ho, P. L. *et al.* Increasing resistance of Streptococcus pneumoniae to fluoroquinolones: results of a Hong Kong multicentre study in 2000. *J Antimicrob Chemother* **48**, 659-665, doi:10.1093/jac/48.5.659 (2001).

11 Wang, H. *et al.* Antimicrobial susceptibility of bacterial pathogens associated with community-acquired respiratory tract infections in Asia: report from the Community-Acquired Respiratory Tract Infection Pathogen Surveillance (CARTIPS) study, 2009-2010. *Int J Antimicrob Agents* **38**, 376-383, doi:10.1016/j.ijantimicag.2011.06.015 (2011).

12 Perez-Trallero, E. *et al.* Antimicrobial susceptibilities of 1,684 Streptococcus pneumoniae and 2,039 Streptococcus pyogenes isolates and their ecological relationships: results of a 1-year (1998-1999) multicenter surveillance study in Spain. *Antimicrob Agents Chemother* **45**, 3334-3340, doi:10.1128/AAC.45.12.3334-3340.2001 (2001).

13 Dobay, O. *et al.* The first steps towards fluoroquinolone resistance in Hungarian pneumococci. *J Chemother* **18**, 624-627, doi:10.1179/joc.2006.18.6.624 (2006).

14 Echlin, H. *et al.* Pyruvate Oxidase as a Critical Link between Metabolism and Capsule Biosynthesis in Streptococcus pneumoniae. *PLoS Pathog* **12**, e1005951, doi:10.1371/journal.ppat.1005951 (2016).
